# Periodic Accumulation of Regulatory T Cells in the Uterus: Preparation for the Implantation of a Semi-Allogeneic Fetus?

**DOI:** 10.1371/journal.pone.0000382

**Published:** 2007-04-18

**Authors:** Marinos Kallikourdis, Alexander G. Betz

**Affiliations:** Medical Research Council (MRC) Laboratory of Molecular Biology, Cambridge, United Kingdom; New York University School of Medicine, United States of America

## Abstract

**Background:**

Naturally occurring Foxp3^+^regulatory T cells play an important role in the inhibition of an immunological attack of the fetus. As implantation of the fetus poses an immediate antigenic challenge, the immune system has to prepare itself for this event prior to implantation.

**Methodology and Principal Findings:**

Here, we show using quantitative RT-PCR and flow cytometry that regulatory T cells accumulate in the uterus not only during pregnancy, but also every time the female becomes fertile. Their periodic accumulation is accompanied by matching fluctuations in uterine expression of several chemokines, which have been shown to play a role in the recruitment and retention of regulatory T cells.

**Conclusions/Significance:**

The data lead us to propose that every time a female approaches estrus, regulatory T cells start to accumulate in the uterus in preparation for a possible implantation event. Once pregnancy is established, those regulatory T cells that have seen alloantigen need to be retained at their site of action. Whilst several chemokines appear to be involved in the recruitment and/or retention of regulatory T cells during estrus, in pregnancy this role appears to be taken over by CCL4.

## Introduction

Naturally occurring Foxp3^+^regulatory T cells play an important role in the prevention of legitimate immune responses that would have deleterious effects on the organism if left unchecked. They prevent autoimmunity [1] as well as an overreaction to commensal bacteria [2] and chronic infection [3]. More recently we have demonstrated that they also mediate maternal tolerance to the fetus. In the absence of regulatory T cells the maternal immune system recognises the semi-allogeneic fetus as foreign and attacks it [4]. Pregnancy itself induces a systemic expansion of the pool of regulatory T cells that starts already prior to implantation and is independent of exposure to alloantigen [4]. Those regulatory T cells that have experienced antigen turn into CCR5^+^effector regulatory T cells, which preferentially accumulate in the gravid uterus [5]. Whether the exposure to fetal antigen occurs in the uterus itself or in a draining secondary lymph organ is unclear. However, interference with the accumulation of effector regulatory T cells at their site of action severely impedes their function [5]–[7]. A hint that the accumulation of regulatory T cells in the gravid uterus is of equal importance in human pregnancies comes from the finding that the number of decidual regulatory T cells in spontaneous abortions is dramatically decreased [8].

The implantation of the fetus expressing paternal transplantation antigens represents a strong antigenic insult, which should lead to an immediate immunological response. The most effective way for the immune system to deal with this problem is to stop such an anti-fetal response at its initiation. Thus one might expect that mechanisms have evolved that prepare the uterus for a possible implantation event. One way to achieve this would be by increasing the likelihood of regulatory T cells encountering paternal alloantigen, by systemic expansion of their number towards estrus [9] and by increasing their presence in the uterus in preparation for implantation. As the paternal alloantigen cannot be known to the immune system prior to implantation both are likely to be antigen independent events. Following implantation one might expect those regulatory T cells that recognize paternal alloantigen to be preferentially retained. Indeed, antigen-experienced CCR5^+^effector regulatory T cells accumulate in the uterus during pregnancy [5].

Here we demonstrate that the number of regulatory T cells present in the uterus is subject to periodic fluctuations. Every time the female approaches estrus regulatory T cells accumulate in the uterine tissue. This fluctuation goes hand in hand with a similar rise and fall in the expression levels of several chemokines that have been shown to be involved in the recruitment and/or retention of regulatory T cells. However, only one of these chemokines, CCL4, remains highly elevated during pregnancy. This concurs with a switch from the accumulation of all regulatory T cells to preferential accumulation of antigen experienced CCR5^+^effector regulatory T cells [5]. Thus we suggest that every time a female becomes fertile the uterus prepares itself by recruiting regulatory T cells even prior to an implantation event.

## Results

### Periodic accumulation of regulatory T cells in the uterus

Regulatory T cells accumulate in the gravid uterus [4]. This can be measured as an increase in the level of Foxp3 mRNA in the uteri from pregnant mice over that of non-pregnant mice. Whilst Foxp3 levels in the uteri of non-pregnant mice are consistently lower, we observed substantial mouse-to-mouse variation. This led us to examine whether the number of regulatory T cells in the uterus fluctuates with the estrus cycle. We could distinguish the four phases of estrus by the variations in the abundance of the cell types present in vaginal lavages. Di-estrus is characterized by the presence of leukocytes and epithelial cells. This is followed by pro-estrus, which can be identified based on the presence of epithelial cells that are close to cornification but still have visible nuclei and cornified epithelial cells. Lavages taken during estrus contain exclusively cornified epithelial cells. The return of leukocytes amongst the cornified cells is characteristic of met-estrus [10].

**Figure 1:**
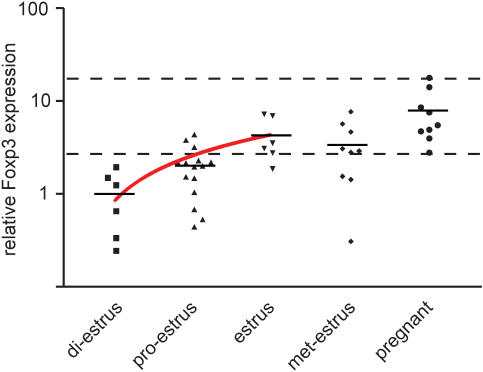
Variation of Foxp3 expression in the uterus during the mouse estrus cycle and pregnancy. Relative levels of Foxp3 were determined by quantitative RT-PCR performed on total uterine mRNA. Uteri were isolated from non-pregnant or pregnant mice at E10.5 (mid-gestation). The estrus cycle phase of non-pregnant mice was determined by vaginal smear and mice were categorised into 4 groups, according to their estrus cycle phase. Foxp3 levels for the 4 phases are shown in chronological order and compared to the levels in pregnant mice. Each point corresponds to one animal. Horizontal bars represent the mean of each data set. Dotted lines indicate the maximal and minimal values detected for pregnant mice. The red line indicates the linear trend (linear regression, displayed on logarithmic scale) for the phases approaching estrus. The experiment was performed on 37 non-pregnant and 10 pregnant mice. HPRT mRNA expression levels were used for normalisation. PCR assays were performed in triplicate.

In order to obtain an approximation of the number of regulatory T cells in the uterus at the various phases of the estrus cycle, we measured the level of Foxp3 mRNA in whole uterine tissue by quantitative RT-PCR (**Fig. 1**). We found that the uterine Foxp3 levels were lowest in di-estrus. There was a statistically significant increasing trend (p = 0.0005, post test for linear trend) in Foxp3 levels from di-estrus to estrus. The uterine Foxp3 levels at estrus and in pregnant mice were significantly elevated compared to the levels observed at di-estrus (p = 0.0073 and p = 0.0065 respectively; t test). These results show that regulatory T cells periodically accumulate in the uterus, peaking during the estrus and then declining until the next approach of estrus. If, however, the mouse becomes pregnant, the level of Foxp3 remains high, reflecting a more long-term accumulation of these cells in the uterus. As we did not correct for variations in uterine size, the levels of Foxp3 might be an underestimate of regulatory T cell numbers during estrus as uteri approaching this phase are substantially enlarged. The same applies in the case of pregnancy. Thus the periodic accumulation of regulatory T cells might be even more pronounced than our data suggest.

### Chemokine expression in the uterus

Several chemokines have been reported to be expressed in the uterus at the various stages of the estrus cycle and during pregnancy. As we are mainly interested in the recruitment and retention of regulatory T cells to the uterus we restricted our analysis to CCL3, CCL4, CCL5, CCL22 and CX3CL1, all of which have been implicated in the regulation of T cell trafficking. CX3CL1 has been shown to be expressed in glandular epithelial cells [11] and decidual stroma [12]. It is thought to be involved in the adhesion of T cells to endothelial cells [13] and its receptor, CX3CR1 can be detected on regulatory T cells [14]. CCL22 is the ligand for CCR4 [15] and has been shown to recruit both activated T cells [16] and regulatory T cells [17], [18]. It is expressed by the uterine epithelium and decidualized stroma [19]. CCL3, CCL4, CCL5 are all ligands of CCR5, which is expressed on both pro-inflammatory effector T cells [5], [20] and effector regulatory T cells [5]. These chemokines are expressed by glandular epithelium, endothelium and stromal cells [12], [19], [21]–[23].

To examine variations in the uterine expression of these chemokines during the estrus cycle and pregnancy, we measured the mRNA levels of the same set of uteri used for the Foxp3 analysis. In about half of the samples the mRNA levels for CCL5 was below the limit of detection **(data not shown)** hindering a detailed statistical analysis. All the other chemokines exhibited a statistically significant rising trend from di-estrus to estrus (CCL3: p = 0.01, CCL4: p = 0.019, CCL22: p = 0.017 and CX3CL1: p = 0.004; post test for linear trend).

**Figure 2:**
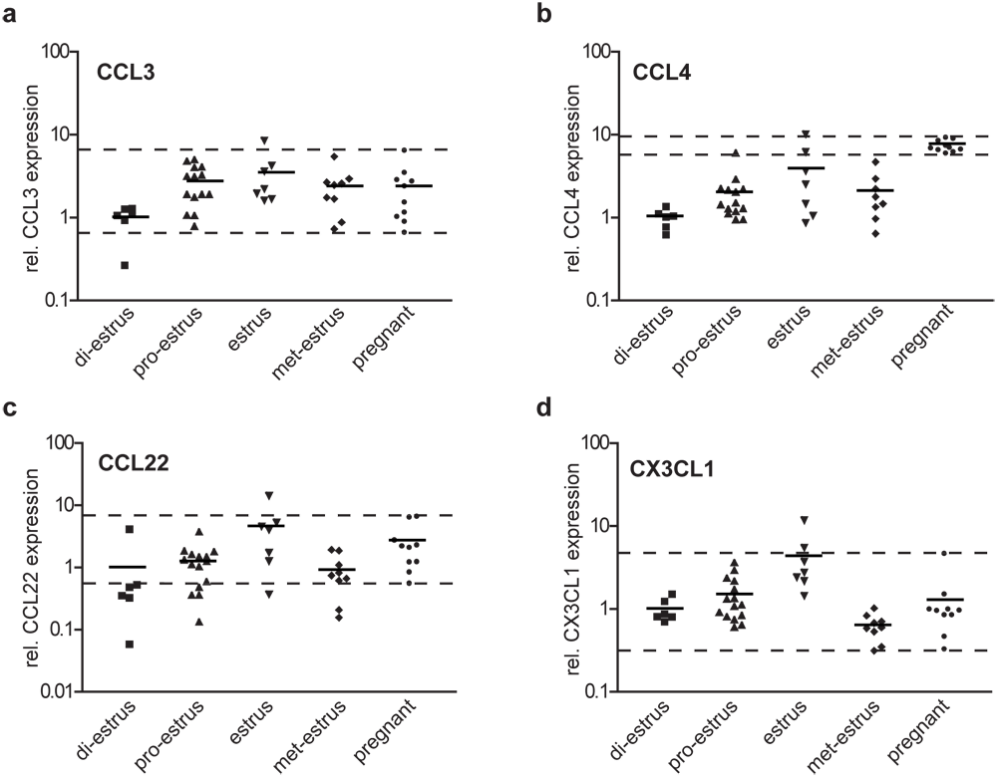
Variation of chemokine expression in the uterus during the mouse estrus cycle and pregnancy. Relative levels of (a) CCL3, (b) CCL4, (c) CCL22 and (d) CX3CL1 were determined by quantitative RT-PCR performed on total uterine mRNA, normalised to HPRT on the same sample set used in Fig. 1. Levels for the 4 estrus cycle phases are shown in chronological order and compared to the levels in pregnant mice. Each point corresponds to one animal. Horizontal bars represent the mean of each data set. Dotted lines indicate the maximal and minimal values detected for pregnant mice.

**Figure 3:**
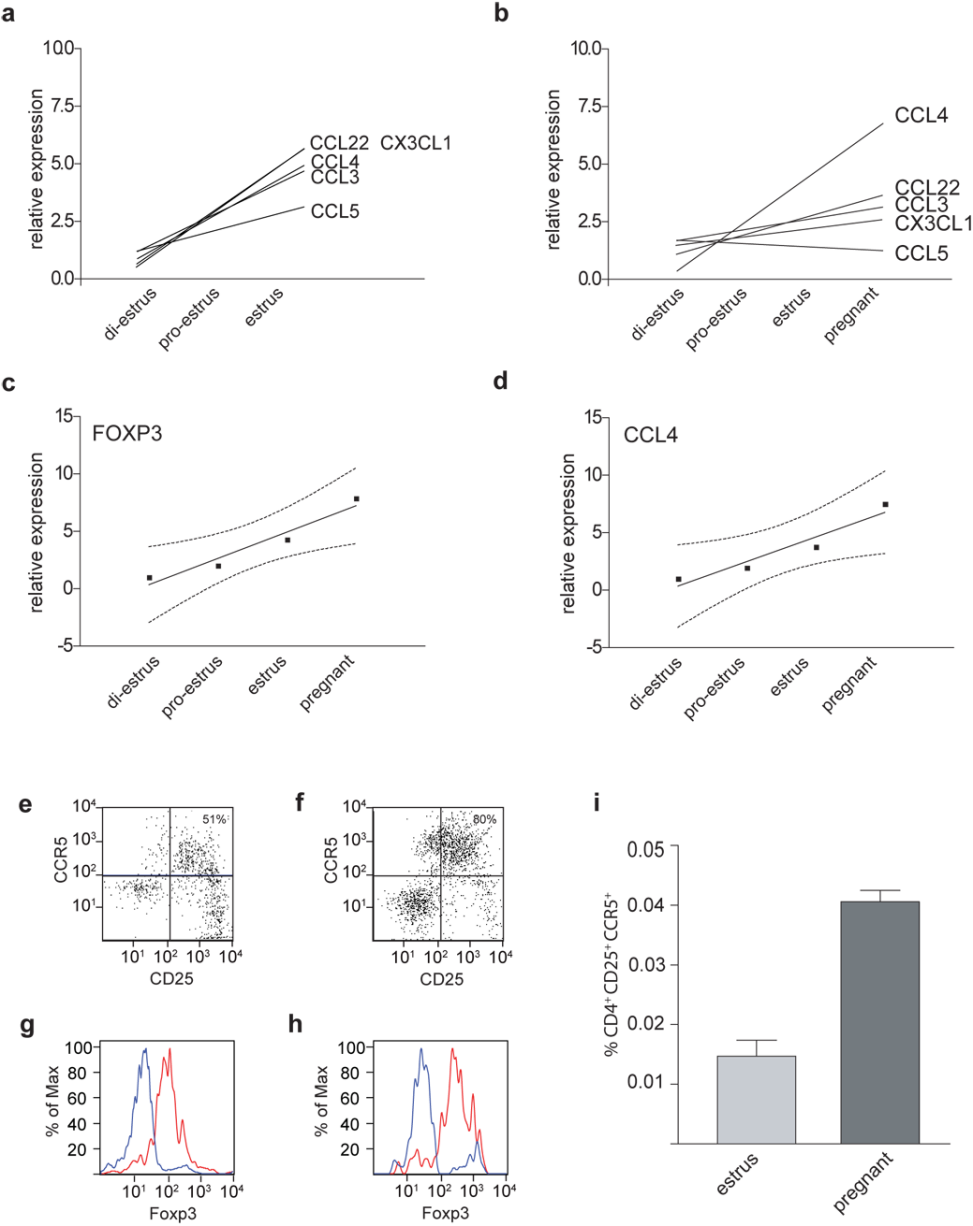
Chemokine fluctuation approaching estrus and pregnancy. Linear trends for the fluctuation of chemokine expression levels (same sample set used in Figs. 1 and 2) during (a) the phases approaching estrus (diestrus>pro-estrus>estrus) and (b) the phases approaching estrus and pregnancy. (c,d): Linear trends for the fluctuation of (c) Foxp3 and (d) CCL4 expression levels during the phases approaching estrus and pregnancy. Dots represent the mean of each data set. Dotted curves represent the 95% confidence interval of the trend line. (e–i) CCR5^+^regulatory T cells in the uterus of mice at estrus or at mid-gestation. n = 3 for estrus, each sample pooled from 3 mice; n = 5 for pregnant, one mouse per sample. Uterine cells were isolated from C57BL/6 mice (e,g) at estrus or (f,h) at E10.5 (mid-gestation) after allogeneic mating and analyzed by FACS for the expression of (e,f) CD4, CD25 and CCR5 or (g,h) CD4, CD25 and Foxp3. (e,f) Plots show CD25 and CCR5 surface expression for all CD4^+^gated cells. Number in top right corner denotes the percentage of CCR5^+^cells amongst CD4^+^CD25^+^cells. 1×10^6^ ‘total’ uterine cells collected; all CD4^+^cells shown. (g,h) Histograms show intracellular Foxp3 stain in (red) CD4^+^CD25^+^or (blue) CD4^+^CD25^−^ cells. Representative samples are shown. (i) Uterine cells from C57BL/6 mice at estrus or at E10.5 of allogeneic pregnancy were analyzed by FACS. The percentage of CD4^+^CD25^+^CCR5^+^cells amongst all uterine cells is shown.

Whilst the expression of all these chemokines was elevated during estrus compared to di-estrus **(Fig. 2a–d)**, with similar linear trends **(Fig. 3a)**, only CCL4 retained a statistically significantly elevated expression (p<0.0001; t-test) during pregnancy **(Fig. 2b)**. This is reflected in the steep slope of the linear trend from di-estrus to pregnancy **(Fig. 3b)**, which is highly significant for CCL4 (p<0.0001, post test for linear trend).

### Accumulation of effector regulatory T cells in the gravid uterus

There is a direct correlation between the level of uterine CCL4 expression and the presence of regulatory T cells as reflected by the Foxp3 level. This is highlighted by the almost identical rising trends from di-estrus to pregnancy **(Fig. 3c and d)**, which in both cases are highly significant (p<0.0001, post test for linear trend). The shift from the elevated expression of multiple chemokines during estrus to the elevated expression of only CCL4 during pregnancy **(Fig. 2)** might cause a change in the type of cell recruited and/or retained. Indeed, we found that during estrus only half of the regulatory T cells in the uterus were CCR5^+^ **(Fig. 3e)** whereas we regularly found more than 70% of regulatory T cells in the gravid uterus expressing CCR5^+^ **(Fig. 3f)**. Furthermore, the total number of CCR5^+^regulatory T cells in the uterus as a proportion of all uterine cells increases significantly **(Fig. 3i)** (p = 0.036; non parametric test). Both, at estrus and E10.5 of pregnancy, over 90% of CD4^+^CD25^+^cells are Foxp3^+^ **(Fig. 3g and h)**, confirming that the vast majority of the cells are indeed regulatory T cells rather than freshly induced proinflammatory effector T cells. Considering that the uterus also increases substantially in size during pregnancy, the increase in CCR5^+^regulatory T cells is even more marked.

**Figure 4:**
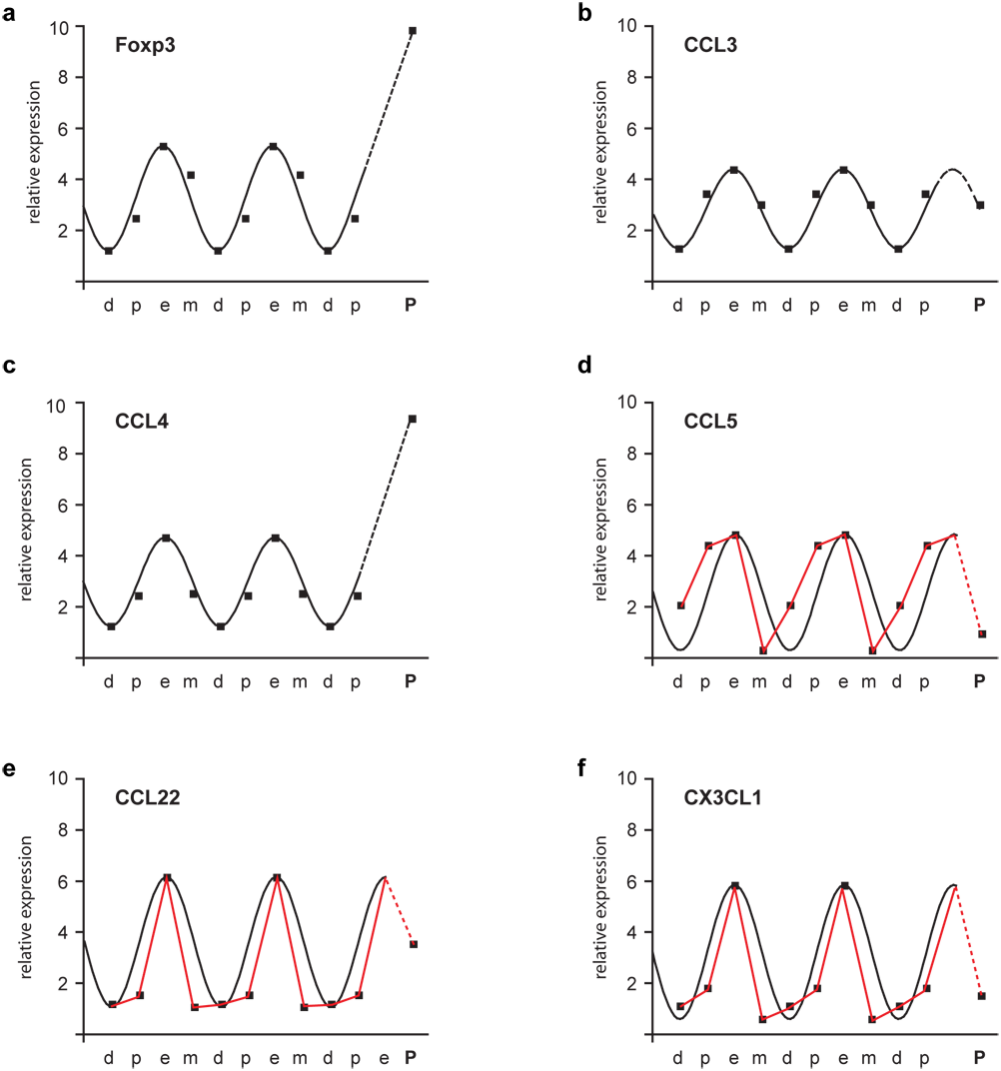
Foxp3 and chemokine expression level fluctuations during the estrus cycle and pregnancy. Plots of the variation of the mean relative expression of Foxp3, CCL3, CCL4, CCL5, CCL22 and CX3CL1 during the estrus cycle phases and pregnancy (same sample set used in Figs. 1 and 2). Estrus cycle phases are arranged in chronological order: (d) diestrus>(p) pro-estrus>(e) estrus>(m) met-estrus>(d) diestrus etc. After 2 full cycles, a cycle were estrus is followed by (P) pregnancy is shown. Dots represent the mean of each data set. Sinusoidal functions (black lines) were fitted to the estrus cycle data (see methods). In the cases where the sinusoidal function did not fit the data, a line connecting the means is shown (red line). Dotted lines join the mean of the estrus phase of the last cycle with the mean of the levels detected during pregnancy.

When plotted in temporal sequence, the fluctuations in Foxp3, CCL3, CCL4 fit a sinusoidal curve peaking at estrus and with a minimum at di-estrus **(Fig. 4a–c)**. It is noteworthy that this is not the case for the other three chemokines examined **(Fig. 4d–f)**. This excludes that the fluctuations observed are a simple reflection of fluctuations in HPRT levels, which were used for normalisation of the samples.

## Discussion

The fetus represents a semi-allograft and thus a strong antigenic insult. Given the importance of placental implantation for mammalian propagation this is far from an ideal scenario from an immunological perspective. Indeed, it might be the reason why regulatory T cells have evolved in the first place [24]. The expansion of the regulatory T cell pool during pregnancy starts before implantation [4]. This might be the way by which the immune system attempts to enhance the likelihood that a particular set of paternal alloantigens can be detected by regulatory T cells. According to this hypothesis, regulatory T cells first expand in number so as to increase the chance that paternally-derived alloantigens will be recognized. Once this has occurred, the emphasis switches to the retention of those regulatory T cells that have been activated by the alloantigen, at their site of action [6], [7]. In the case of pregnancy this is most likely the uterus [5].

Our data shows that the uterine expression of all chemokines analyzed displays periodic variation peaking around estrus **(Fig. 4)**. This is in agreement with the notion that chemokines are involved in the remodelling of the uterine architecture and cell composition during the estrus cycle [19], [25]. All these chemokines are also involved in T cell recruitment and retention [15]. Interestingly, all three ligands (CCL3, CCL4, CCL5) for CCR5 are expressed at an elevated level during estrus. CCR5 is expressed by the effector arm of both pro-inflammatory and regulatory T cells [5], [20]. It is tempting to speculate that this can lead to an accumulation of both cell types during estrus. Under favourable health conditions the balance between regulatory T cells to pro-inflammatory T cells would be in favour of the regulatory cells **(Fig. 3 g and h)** [4], [8] and indeed we can measure this in the form of a significant increase in Foxp3 mRNA in the uterus **(Fig. 1)**.

In contrast, during an infection pregnancy might become an undesirable high-risk event. This is particularly the case when the urogenital tract is affected [26]. In this scenario the prevention of an implantation of the fetus might be of advantage for the mother [27]. The recruitment of abundant pro-inflammatory CCR5^+^effector T cells to the uterus would be likely to lead to a loss of tolerance. Indeed, an experimentally induced increase in pro-inflammatory CCR5^+^effector T cells has been demonstrated to cause an increase in abortions [20].

All the chemokines analyzed have the potential to influence the accumulation of regulatory T cells in the uterus during estrus, as their corresponding receptors are expressed on regulatory T cells [5]–[7], [14], [17]–[18]. However, CCL4 is the only chemokine with elevated expression during pregnancy. Given the fact that alloantigen-experienced effector regulatory T cells express CCR5 [5]–[6], it is likely that CCL4 is responsible for the retention of these cells in the gravid uterus [5].

Our data show that every time a female becomes fertile regulatory T cells start to accumulate in the uterus. Regulatory T cells may be actively recruited to the uterus in order to be exposed to paternal alloantigen upon implantation. Those regulatory T cells that recognize paternal alloantigen become activated, up-regulate CCR5 and thus are retained in the uterus. Alternatively the expression of these chemokines might be in preparation for the passive accumulation by preferential recruitment and/or retention of those regulatory T cells that encounter paternal alloantigen in the draining lymph nodes, become activated and up-regulate CCR5. In this scenario the accumulation of regulatory T cells during estrus might simply be a side effect of the uterus getting ready to retain those regulatory T cells that will become activated by alloantigen. Whether the cells see alloantigen in the uterus itself or get activated in the secondary lymphoid organs will be subject of future investigation.

## Materials and Methods

### Experimental animals

Mice were kept in specific pathogen-free conditions. All mice used in the quantitative PCR studies were of BALB/c×C57BL/6 F1 background. Mice were identified as being in di-estrus, pro-estrus, estrus or met-estrus by Giemsa (Sigma-Aldrich, UK) staining of smears of vaginal lavages, as per manufacturer's instructions [10]. C57BL/6 females were used for the ‘estrus versus pregnant’ comparison in which the pregnant mice were allogeneically mated by BALB/c males.

### Preparation of uterine tissue

Whole uteri were separated from surrounding tissue (including fetuses/placenta in the case of the pregnant mice). For flow cytometric analysis the uteri were digested in collagenase A (Roche Diagnostics, UK) for 15min according to manufacturer's instructions, washed in PBS and then gently pushed through a (60µm diameter pore size) cell strainer (BD, UK) before being resuspended in PBS/2%FCS for counting and staining. For RNA purification, the tissue was immersed in liquid nitrogen, pulverized and re-suspended in RNA lysis buffer. Total RNA was prepared using RNeasy kit (Qiagen, UK) incl. on-column DNase digestion, according to manufacturer's instructions.

### Quantitative real-time RT-PCR

cDNA was synthesized using SuperscriptII RT (Invitrogen, UK), with random hexamer primers, as per manufacturer's instructions. Real time PCR was performed using Taqman universal PCR master mix (Applied Biosystems, UK) and primers and a fluorescent TaqMan probe specific for each gene analyzed or HPRT (used for normalization). An ABI prism 7700 sequence detection system was used for 45 cycles of PCR. PCRs were set up at least in triplicate. The sequences were: Foxp3 primers as in [28], CCL3-primers: 5′-CCA AGT CTT CTC AGC GCC ATA-3′ and 5′-GAT GAA TTG GCG TGG AAT CTT C-3′; CCL3-probe: 5′-**FAM**-AGC TGA CAC CCC GAC TGC CTG C-**TAMRA**-3′; CCL4-primers: 5′-TGC TCG TGG CTG CCT TCT-3′ and 5′-CTG CCG GGA GGT GTA AGA GA-3′; CCL4-probe: 5′-**FAM**-TGC TCC AGG GTT CTC AGC ACC AAT G-**TAMRA**-3′; CCL5-primers: 5′-CTG TCA TCG CTT GCT CTA GTC CTA-3′ and 5′-CGG ATG GAG ATG CCG ATT T-3′; CCL5-probe: 5′-**FAM**-ATC CCC TAC TCC CAC TCC GGT CCT G-**TAMRA**-3′, CCL22-primers 5′-CAG GTC CCT ATG GTG CCA AT-3′ and 5′-AAC GTG ATG GCA GAG GGT G-3; CCL22-probe: 5′-**FAM**-AAG ACA GTA TCT GCT GCC AGG ACT ACA TCC G-**TAMRA**-3′ HPRT-primers: 5′-TTA AGC AGT ACA GCC CCA AAA TG-3′ and 5′-CAA ACT TGT CTG GAA TTT CAA ATC C-3′ and HPRT probe: 5′-**VIC**-CCT TTT CAC CAG CAA GCT TGC AAC CTT A-**TAMRA**-3′. CX3CL1 mRNA quantitation was performed using a CX3CL1 TaqMan Gene Expression Assay (Mm00436454) (Applied Biosystems, UK).

### Flow cytometric analysis

Cells were labelled with fluorescein isothiocyanate (FITC)-anti-CD25 (7D4), phycoerythrin (PE)-anti-CCR5 (C34-3448), (Cy)-anti-CD4 (L3T4)(H129.19) and analyzed on a FACScan. Phycoerythrin (PE)-Rat IgG2c was used as isotype control for the CCR5 stain (all antibodies were purchased from BD, UK). Foxp3 intra-cellular staining was performed using (PE)-anti-Foxp3 kit (eBiosciences, US) after permeabilisation of the surface stained cells according to manufacturer's instructions.

### Statistical analysis

All statistical analysis was performed using the Prism package (Graphpad, US). Trendlines were fitted by linear regression. Sinusoidal functions of the fluctuations during the estrus cycle were constrained by assuming the period to be equal to 1 full estrus cycle, the amplitude to be equal to half the maximal variation of the mean value during one full cycle, and the baseline to be equal to the lowest value of the mean during one full cycle plus one amplitude
